# Mucormycosis in renal transplant recipients: review of 174 reported cases

**DOI:** 10.1186/s12879-017-2381-1

**Published:** 2017-04-18

**Authors:** Yan Song, Jianjun Qiao, Gaffi Giovanni, Guangjun Liu, Hao Yang, Jianyong Wu, Jianghua Chen

**Affiliations:** 1 Kidney Disease Center, The First Affiliated Hospital, College of Medicine, Zhejiang University, No. 79 Qingchun Road, Hangzhou, 310003 Zhejiang People’s Republic of China; 2 Department of Dermatology, The First Affiliated Hospital, College of Medicine, Zhejiang University, Hangzhou, People’s Republic of China; 3 Azienda Ospedaliero-Universitaria Ospedali Riuniti, Ancona, Italy

**Keywords:** Mucormycosis, Renal transplant recipient

## Abstract

**Background:**

Mucormycosis is a highly lethal fungal infection especially in immunocompromised individuals.

**Methods:**

In order to review the epidemiology, diagnosis, and treatment of mucormycosis in renal transplant recipients we searched publications of mucormycosis cases in renal transplant recipients in PUBMED database up to December 2015.

**Results:**

A total of 174 cases in renal transplant recipients were included in this review. Most of the cases (76%) were male. Major underlying diseases were diabetes mellitus (43.1%). Rhinocerebral was the most common site of infection (33.3%). *Rhizopus* species was the most frequent fungus (59.1%) in patients with pathogen identified to species level. The mortality rates of disseminated mucormycosis (76.0%) and graft renal (55.6%) were higher than infection in other sites. The overall survival in patients received surgical debridement combined with amphotericin B/posaconazole (70.2%) was higher than those who received antifungal therapy alone (32.4%), surgery alone (36.4%) or without therapy (0%) (*p* < 0.001). The overall survivals in patients receiving posaconazole and lipid amphoterincin B were higher than that receiving deoxycholate formulation (92.3% and 73.4% vs 47.4%).

**Conclusions:**

Mucormycosis is a severe infection in renal transplant recipients. Surgical debridement combined with antifungals, especially liposomal amphotericin B and posaconazole, can significantly improve patient’s overall survival.

**Electronic supplementary material:**

The online version of this article (doi:10.1186/s12879-017-2381-1) contains supplementary material, which is available to authorized users.

## Background

Mucormycosis has become an increasingly emerging life-threatening invasive fungal infection especially in immunocompromised patients in the past decades, including patients with hematological malignancies and hematopoietic stem cell transplant recipients, solid organ transplant recipients, patients with diabetes mellitus, surgical patients, patients with burns, injection drug users, trauma patients, and those undergoing deferoxamine therapy [1–4]. The infection rates of post-transplant fungal infections were approximately 2–14% [5]. Mucormycosis is associated with the longest duration of hospitalization and the shortest 2-year survival in renal transplant (RT) patients, although it comprises only 2–6% of invasive fungal infection [6].

In order to identify the epidemiology, risk factors, prognosis of mucormycosis and outcome in renal transplant recipients, we examined all published cases of mucormycosis in RT patients. We assessed the demographic data, clinical, histopathological and microbiological findings, laboratory tests, management and outcomes of the RT patients with mucormycosis.

## Methods

### Literature search

Our goal was to discover the demographic characteristics, the underlying conditions, the site and pattern of infection, microbiologic and histopathologic findings, management and outcomes in RT patients with mucormycosis by review published cases. The literature search was limited to case or case series of mucormycosis in RT patients reported in English language. The PubMed database was searched for all mucormycosis case until December 2015 using the following key words: renal transplant, renal transplantation, kidney transplant, kidney transplantation, zygomycosis, mucormycosis, phycomycosis, *Absidia (Mycocladus)*, *Apophysomyces*, *Cokeromyces*, *Cunninghamella*, *Mucor*, *Rhizopus*, *Rhizomucor*, *Saksenaea*, and *Syncephalastrum*.

### Case selection criteria

Studies were eligible for inclusion if they reported a case or case series of mucormycosis in RT patients. Only cases which documented the following data were included in the review: age and sex of patients, anatomical location of infection, diagnostic methods, therapeutic strategy (including antifungal drugs and surgical therapy) and outcome. Zygomycosis caused by *Entomophthorales* was not included in this review.

### Data extraction

From each case, we extracted publication year, sex and age of patients, primary underlying condition, duration from transplantation to disease onset, allograft rejection events and the primary site of infection at time of diagnosis, fungal culture, histopathology, antifungal therapy, surgical therapy and outcomes.

### Statistical analysis

The association between potential risk factors and mortality were performed by using univariate analyses. Logistic regression analysis was used for multivariate analysis of variables found to be significant with univariate analysis. The variables were considered for inclusion in a multivariate model if they have a *p* < 0.20 on univariate analysis. Values of 2-tailed *p* < 0.05 were considered statistically significant. All statistical analyses were performed using the Statistics Package for Social Science (SPSS) version 17.0.

## Results

**Fig. 1:**
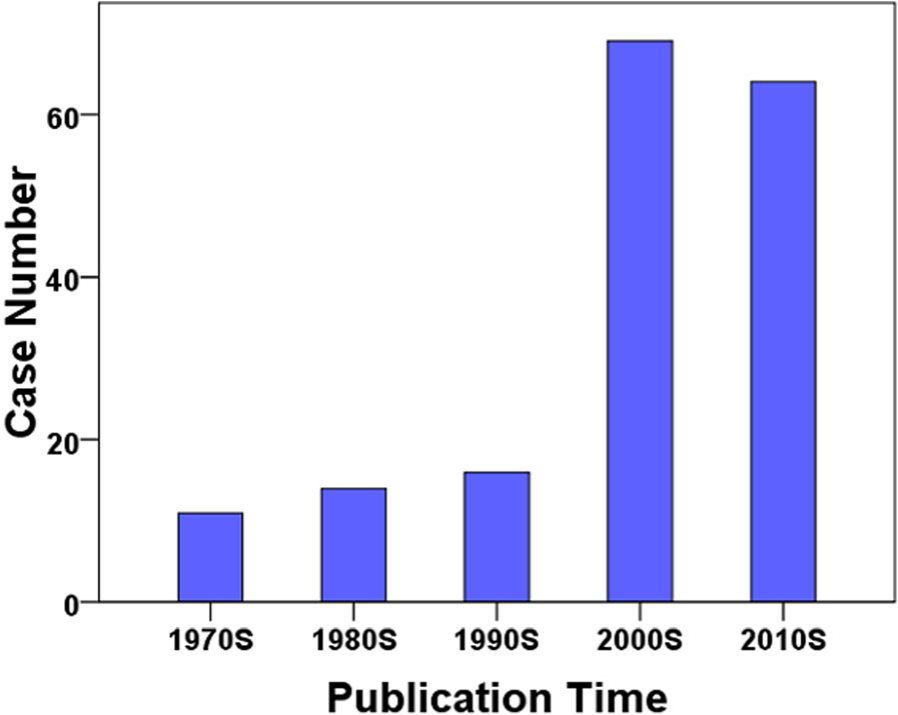
Case numbers of mucormycosis since the 1970s

Using our search strategy, it was yielded 253 articles reporting mucormycosis in RT patients. Abstracts and/or full texts were reviewed by two authors. One hundred and thirty-two articles were excluded because data of age, sex, therapy or outcome were not available. Entomophthoromycosis were exclude because the prognosis of diseases caused by *Entomophthorales* and *Mucorales* were different. Articles reported in non-English language were also excluded. Finally, a total of 174 cases of mucormycosis in renal transplant recipients were identified in 123 articles published from 1970 to 2015 (Additional file 1). The reporting of mucormycosis in renal transplant recipients was increasing during the study period (Fig. 1). One hundred and thirty-four cases were published after 2000, accounting for 77% of the patients (Fig. 1).

### Demography and medical history

The demographic characteristics and underlying clinical conditions of the RT recipients with mucormycosis are summarized in Table 1. The average age of the 174 patients was 45.9 years (range, 11–70 years), with male representing the majority (76%). The most common underlying diseases was diabetes mellitus (43.1%). Nine of them were type 1 diabetes, 6 were type 2 diabetes, and 10 were post-transplant diabetes. In 50 of them the diabetes type was not described. The overall mortality of patients with diabetes mellitus was 33.3%. Other underlying conditions including surgery, dental extraction, HIV infection, malignancy, trauma, and deferoxamine therapy. Of note, thirty (25.1%) patients had no special medical history apart from RT. The median time duration from RT to establishing the diagnosis of mucormycosis was 2.5 months (range, 2 days-20 years).

**Table 1:** Demographic and clinical characteristics of 174 renal transplant recipients with mucormycosis

Feature	All patients	Proportion of patients who died
Mean age, y	45.9 (range 11–70)	
Gender		
Male	133(76%)	58/133 (43.6%)
Female	41(24%)	16/41 (39.0%)
Underlying conditions		
No	30/174 (25.1%)	15/30 (50.0%)
Diabetes	75/174 (43.1%)	25/75 (33.3%)
Other conditions^a^	19/174 (3.4%)	7/19 (36.8%)
Not reported	50/174 (28.7%)	27/50 (54.0%)
Transplanted organs		
Kidney alone	162/174(93.1%)	71/162(43.8%)
Kidney combined with other organs	12/174(6.9%)	9/12(75.0%)
Anti-allograft rejection therapy		
Yes	33/174(18.97%)	12/33(36.4%)
No	16/174(9.1%)	7/16(43.8%)
Not reported	125/174(71.8%)	55/125(44.0%)
Induction therapy		
Yes	33/174(19.0%)	12/33(36.4%)
No	16/174(9.2%)	7/16(43.8%)
Not reported	125/174(71.8%)	55/125(71.8%)
Reduce immunosuppressive drugs		
Yes	98(56.3%)	38/98(38.8%)
No	6(3.4%)	2/6(33.3%)
Not reported	70(40.2%)	34/70(48.6%)
Diagnostic methods		
Histopathology only	72/174(41.4%)	33/72(45.8%)
Culture only	25/174(14.4%)	9/25(36.0%)
Histopathology and culture	77/174(44.3%)	32/77(41.6%)

^a^Including surgery, dental extraction, HIV infection, malignancy, trauma, and deferoxamine therapy

One hundred and sixty-two patients received RT alone, the other 12 patients received combined solid organ transplantation, including the pancreas, liver, and heart. Although the mortality in combined organ transplantation recipients was higher than that in recipients received kidney transplantation alone (75.0% vs 43.8%), combined transplantation was not a risk factor for mortality due to mucormycosis (unadjusted OR, 0.22; 95% CI, 0.11–1.64). Acute allograft rejection occurred in 52(29.9%) patients, 33(63.5%) of them received induction therapy. Neither anti-allograft rejection therapy nor induction therapy was risk factor of mortality due to mucormycosis (*p* > 0.20). The anti-rejection drugs were discontinued or reduced in 98(56.3%) patients.

### Sites of infection

According to the clinical presentation and anatomic localization, there were 7 major clinical forms of mucormycosis infection: (1) rhinocerebral, (2) pulmonary, (3) cutaneous, (4) gastrointestinal, (5) graft kidney (6) disseminated, and (7) other uncommon sites (Table 2). Disseminated infection was defined as infection at 2 noncontiguous sites. The types of infection by site at the time of diagnosis in our review were shown in Table 2. Overall, rhinocerebral mucormycosis is the most common form (33.3%), followed by pulmonary (25.9%), disseminated (14.4%), transplanted kidney (11.5%), cutaneous (7.5%), gastrointestinal (5.7%), peritoneal (1.1%) and artery stent (0.6%). The mortality of mucormycosis in RT recipients was varied with the site of infection: 76% of patients with disseminated mucormycosis, 42.2% with pulmonary infection, and 55.6% with renal graft infection (Table 2).

**Table 2:** Infection patterns among 174 renal transplant recipients with mucormycosis, 74 of whom died

Type of infection	Proportion of all patients	Number of patients who died/total number (%)
Disseminated	25 (14.4%)	19/25 (76.0%)
Pulmonary	45 (25.9%)	19/45(42.2%)
Gastrointestinal	10 (5.7%)	4/10 (40%)
Cutaneous	13 (7.5%)	3/13 (23.1%)
Graft kidney	20 (11.5%)	11/20 (55.6%)
Rhinocerebral	58 (33.3%)	18/58 (31.0%)
Peritoneal	2(1.1%)	0/2(0%)
Artery stent	1 (0.6%)	0/1 (0%)
Total	174 (100%)	74/174(42.5%)

Of the 58 patients with rhinocerebral infection, the initial symptoms of 33 (56.9%) patients were headache and 25 (43.1%) patients had a history of diabetes or hyperglycemia. The mortality of disseminated mucormycosis was much higher than that of localized diseases (*p* < 0.001, OR = 5.41; 95% CI, 2.04–14.37).

Signs that suggest graft kidney mucormycosis were nonspecific, including fever, abdominal pain, oliguria, and graft dysfunction. However, absence of this findings should not exclude the possibility of mucormycosis. Fever was variable and may be absent in up to half of cases. There was only one case accompany with urinary leak before graft infection.

There were two cases of peritoneal dialysis-associated zygomycete peritonitis after renal transplantation. The patients have nonspecial symptoms, including abdominal pain and cloudy dialysis effluent. Tenckoff dialysis catheter were removed in the two zygomycete peritonitis patients and converted to maintain hemodialysis.

### Secular trends in reported hosts

Our study finds that there was a rising incidence of mucormycosis in RT recipients in the past 5 decades (Fig. 1). The majority cases documented by culture were reported since the 2000s. This could be caused by the advances in diagnostic techniques.

### Microbiologic and histopathologic findings

All patients were confirmed as mucormycosis by histological examination and/or fungal culture. Histological examination was performed in 160 cases. 149 (85.6%) of which revealed typical hyphae. Of the 174 cases, 77 (44.3%) were diagnosed by both histopathology and culture, 25(14.4%) by culture only, 72 (41.4%) by histopathology only (Table 1). In 88 patients pathogenic zygomyectes were identified to species level (Table 3). The most frequent pathogen was *Rhizopus* species (59.1%), followed by *Mucor* species (13.6%) and *Cunninghamella* species (8.0%).

**Table 3:** Microbiological findings for 88 renal transplant recipients with mucormycosis, 38 of whom died

Isolated zygomycetes	Number (%) of all patients	Number of patients who died/total number (%)
*Mycocladus* (*Absidia*) species	4 (4.5%)	2/4(50%)
*Apophysomyces elegans*	5 (5.7%)	3/5(60%)
*Cunninghamella* species	7 (8.0%)	2/7(28.6%)
*Mucor* species	12 (13.6%)	7/12 (58.3%)
*Rhizomucor* species	6 (6.8%)	3/6 (50%)
*Rhizopus* species	52(59.1%)	19/52 (36.5%)
Not speciated	26 (29.5%)	7/26 (26.9%)
*Rhizopus oryzae*	18 (20.5%)	7/18 (38.9%)
*Rhizopus microsporus*	5 (5.7%)	4/5 (80%)
*Rhizopus rhizopodiformis*	3 (3.4%)	1/3 (33.3%)
Other	2 (2.3%)	0/2 (0%)
Total	88 (100%)	36/88 (40.9%)

### Sex and mucormycosis

**Fig. 2:**
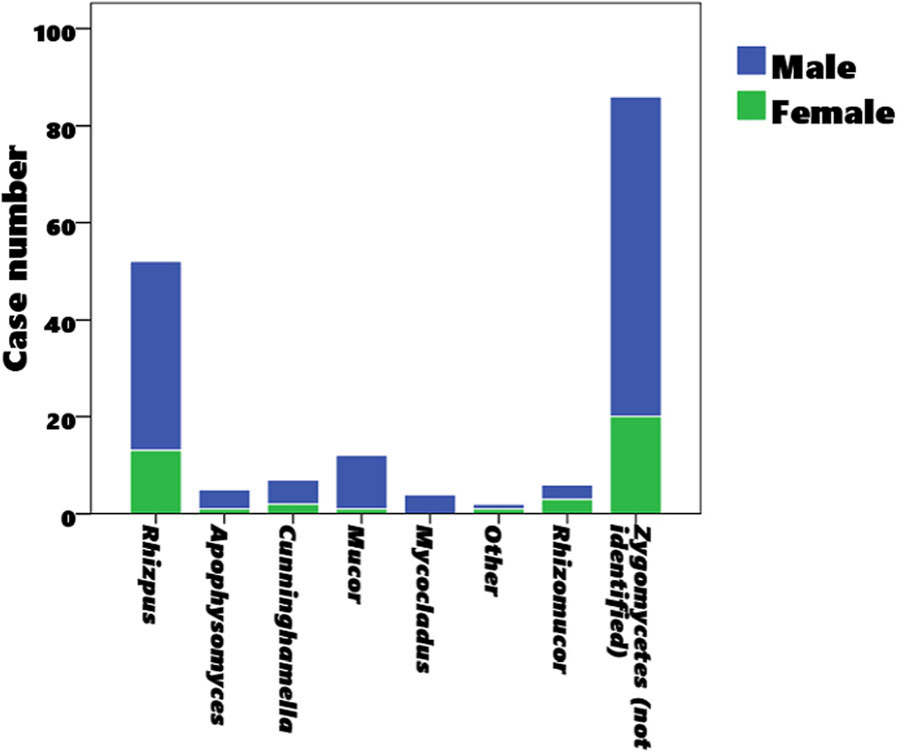
Male to female ratio of pathogenic fungal species in 174 renal transplant recipients with mucormycosis

Most cases of mucormycosis in RT recipients (76%) were male. Infection with *Mycocladus* (100%), *Apophysomyces elegans* (80.0%), *Mucor* (90.9%) and *Rhizopus rhizopodiformis* (100%) was highly associated with male gender (Table 4, Fig. 2).

**Table 4:** Relationship between gender and pathogenic fungal species in 174 renal transplant recipients with mucormycosis

Isolated zygomycetes	Number of male cases/total no. of cases (%)
*Mycocladus* (*Absidia*) species	4/4(100%)
*Apophysomyces elegans*	4/5(80.0%)
*Cunninghamella* species	5/7(71.4%)
*Mucor* species	10/11(90.9%)
*Rhizomucor* species	3/6(50.0%)
*Rhizopus* species	39/52(75.0%)
Not speciated	19/26(73.1%)
*Rhizopus oryzae*	14/18(77.8%)
*Rhizopus microsporus*	3/5(60.0%)
*Rhizopus rhizopodiformis*	3/3(100%)
Other	1/2(50.0%)
Unidentified zygomycetes	66/86(76.7%)
Total	133/174(76.4%)

### Therapy

The treatment strategies included antifungal therapy, surgery, and reduction of immunosuppression (Table 5). One hundred and twenty-one (69.5%) patients received both antifungals and surgery therapy. The survival rate of this group patients (70.2%) was much higher than those who received either antifungal therapy alone (32.4%) or surgery alone (36.4%) (*p* < 0.001). Of the 174 cases reviewed, 155 (89%) received antifungal chemotherapy (Table 5). The survival rate of patients received deoxycholate amphotericin B (37/78, 47.4%), liposomal amphotericin B (47/64, 73.4%) and posaconazole (12/13, 92.3%) therapy was higher than those receiving other antifungals (4/19, 21.1%) or no treatment (0/8, 0%), (*p* < 0.001) (Table 6). The overall survivals in patients receiving posaconazole and lipid amphoterincin B were higher than that receiving deoxycholate formulation (92.3% and 73.4% vs 47.4%) (Table 4).

**Table 5:** Therapy of 174 renal transplant recipients with mucormycosis

Therapy	Patient number	Survived/total number
Amphotericin B formulation		
Deoxycholate	78/174(44.8%)	37/78(47.4%)
Lipid	64/174(36.8%)	47/64(73.4%)
Posaconazole	13/174(7.5%)	12/13(92.3%)
Itraconazole, voriconazole, fluconazole, encanocandins, and no antifungal therapy	19/174(11.0%)	4/19(21.1%)
Surgery alone	11/174(6.3%)	4/11(36.4%)
Surgery and antifungal therapy	121/174(69.5%)	85/121(70.2%)
Antifungal alone	34/174(19.5%)	11/34(32.4%)
None	8/174(4.6%)	0/8(0%)

**Table 6:** Risk factors for mortality of renal transplant recipients with mucormycosis

Variable	OR (95% CI)	*P*
Extent of infection		
Localized	Reference	
Disseminated	5.41(2.04–14.37)	0.001
Organism		
*Rhizopus* spp.	Reference	
Other organisms		>0.1
Diabetes		
No	Reference	
Diabetes	0.43(0.20–0.89)	0.02
Antifungal therapy		
None	Reference	
Amphotericin B deoxycholate	0.30 (0.09–0.97)	0.04
Lipid amphotericin B	0.10(0.03–0.33)	<0.001
Posaconazole alone and combined with other antifungals	0.02(0.00–0.22)	<0.01
Surgery		
Surgery as primary therapy	Reference	
Without surgery therapy	5.83(2.68–12.70)	<0.001

## Discussion

There have been several studies about mucormycosis in patients with diabetes mellitus, [7, 8] and hematologic malignancies [9, 10]. Although there are some studies focus on the epidemiology and risk factors of mucormycosis in solid organ transplant recipients [11, 12], this is the first critical review of the epidemiology, diagnosis, and treatment of mucormycosis in RT patients. We find that the incidence of mucormycosis in RT patients was growing within the past several decades. The growing trend of mucormycosis may be associated with enhanced use of immunosuppressive therapy, prolonged prophylaxis with antifungals lacking activity against zygomycetes, the rising prevalence of diabetes mellitus, advances in diagnostic techniques, increased awareness of clinicians and publication bias.

Our study indicates that there is a higher prevalence of mucormycosis in male RT patients. This finding is consistent with a comprehensive literature review of mucormycosis [3]. While we cannot find the predictor factors in male patients in our critical review. We postulate the sex preference may be owing to the protective role of estrogen, [13] habits and customs, or other differences between male and female.

Mucormycosis remains a seriously threat in patients with diabetes mellitus [2, 7]. Our study also shows that RT patients with diabetes mellitus are prone to develop mucormycosis. However, diabetes mellitus is not an independent risk factor for mortality in RT patients with mucormycosis. This is mainly due to the good control of blood glucose in diabetes patients and the enhanced treatment with statins which are active against some zygomycetes [14, 15].

Although the overall mortality rate in patients received combined solid organ transplantation higher than that in patients received RT alone (75.0% vs 43.8%), combined organ transplantation is not an independent risk factor for mortality (*p* = 0.22). The statistic indifference may be caused by the low number of patients received combined organ transplantation.

Theoretically the use of immunosuppressive therapeutics, especially the induction therapy and the anti-allograft rejection therapy, are risk factors of fungal infection. Immunosuppressive agents can increase patient’s susceptibility to mucormycosis by causing function defects of macrophages and neutrophils and/or by causing drug-induced diabetes [16]. However, we did not find that anti-allograft rejection therapy influences the incidence and the mortality of mucormycosis. This could be explained by the fact that the numbers of patients not receiving anti-allograft rejection treatment (16 patients, 9.1%) and reducing immunosuppressants (6 patients, 3.4%) are very small. For some RT recipients, another reason for this indifferent is that mucormycosis occurred prior to immunosuppressive treatment.

Renal involvement is a rare manifestation of mucormycosis. In RT recipients, graft mucormycosis is relatively common (11.5%). Among RT patients with localized mucormycosis, the mortality rate of graft kidney mucormycosis is the highest (11/20, 55.6%). They were suspected as acute rejection upon clinical signs and symptoms in 20% RT recipients. So, patients who are suspected as rejection should be alert for mucormycosis before anti-rejection therapy.

The overall survival in patients receiving amphotericin B combined with surgery therapy is higher than those receiving amphotericin B alone or surgery alone. Therefore, the first-line therapy for mucormycosis in RT recipients should be amphotericin B lipid formulation combined with surgery debridement. This is consent with the recommendation to general patients [3].

The overall survival in patients receiving lipid amphoterincin B is higher than that receiving deoxycholate formulation. Renal toxicity and is the most serious side effect of amphotericin B. We postulate that the lower overall survival of deoxycholate amphoterin B may be associated with its side effects, which hold back deoxycholate formuation to be used. Posaconazole was a new azole antifungal with activity against zygomycetes. The overall survival is 92% in patients receiving posaconazole therapy, its higher than those receiving amphotericin B. Recent studies also have shown overall success rates of 60–70% with posaconazole as salvage therapy for zygomycetes infection [17, 18]. These encouraging data suggest that posaconazole may represent a prospective drug against mucormycosis.

## Conclusion

In conclusion, mucormycosis is a fungal infection with high mortality in RT patients. In RT recipients with disseminated and graft kidney mucormycosis have the worst prognosis. Surgical debridement combined with antifungals (amphotericin B formulation and posaconazole) can significantly improve patient’s overall survival. The effect of liposomal amphotericin B and posaconazole seems better than amphotericin B deoxycholate against mucormycosis in renal transplant recipients. Clinicians should increase precautions to mucormycosis in RT recipients.

## Additional file

Additional file 1: Published cases of mucormycosis in renal transplant recipients included in this review. (DOCX 105 kb)

## Abbreviation

RT: Renal transplantation
