# Supplementary material for: Mucormycosis in renal transplant recipients: review of 174 reported cases
Source: BMC Infect Dis. 2017 Apr 18;17:283. doi: 10.1186/s12879-017-2381-1 (PMC5395857; doi:10.1186/s12879-017-2381-1)
Supplement: Additional file 1: — Published cases of mucormycosis in renal transplant recipients included in this review. (DOCX 105 kb) [file 12879_2017_2381_MOESM1_ESM.docx]

**Supplementary material**

**Published cases of mucormycosis in renal transplant recipients included in this review**[1-123]

1. Zhu X, Liu H, Wang W, Song S, Jin M, Hu X, Zhang X. Two cases of transplant renal artery thrombosis and spontaneous rupture caused by mucormycosis. Transpl Infect Dis. 2015; 17(3):442-8.

2. Tan M, Gibney EM. Lung mass in a kidney transplant recipient. Am J Transplant. 2015; 15(1):281-2; quiz 3.

3. Talebi-Taher M, Alavi Niakou SN, Javad-Mousavi SA, Vaziri M, Iranpour A, Dehghani M. Pulmonary Mucormycosis in a Patient with Chronic Rejection of Kidney Transplant: A Case Report. Tanaffos. 2015; 14(2):149-52.

4. Nandwani A, Jha PK, Duggal R, Kher V. Invasive gastric mucormycosis and cytomegalovirus infection in an ABO incompatible renal transplant recipient. Indian J Nephrol. 2015; 25(6):373-6.

5. Nam Y, Jung J, Park SS, Kim SJ, Shin SJ, Choi JH, Kim M, Yoon HE. Disseminated mucormycosis with myocardial involvement in a renal transplant recipient. Transpl Infect Dis. 2015; 17(6):890-6.

6. Davuodi S, Manshadi SA, Salehi MR, Yazdi F, Khazravi M, Fazli JT. Fatal cutaneous mucormycosis after kidney transplant. Exp Clin Transplant. 2015; 13(1):82-5.

7. Chi M, Kim HJ, Basham R, Yoon MK, Vagefi R, Kersten RC. Temporal Artery Calciphylaxis Presenting as Temporal Arteritis in a Case of Rhinoorbitocerebral Mucormycosis. Ophthal Plast Reconstr Surg. 2015; 31(5):e132-5.

8. Ville S, Talarmin JP, Gaultier-Lintia A, Bouquie R, Sagan C, Le Pape P, Giral M, Morio F. Disseminated Mucormycosis With Cerebral Involvement Owing to Rhizopus Microsporus in a Kidney Recipient Treated With Combined Liposomal Amphotericin B and Posaconazole Therapy. Exp Clin Transplant. 2014.

9. Stewart JI, D'Alonzo GE, Ciccolella DE, Patel NB, Durra H, Clauss HE. Reverse halo sign on chest imaging in a renal transplant recipient. Transpl Infect Dis. 2014; 16(1):115-8.

10. Rasiah S, Fernandes KD, Sajiv CT, Pawar B. A case of fatal disseminated Apophysomyces elegans infection in a renal allograft recipient. Indian J Nephrol. 2014; 24(1):54-6.

11. Rammaert B, Angebault C, Scemla A, Fraitag S, Lerolle N, Lecuit M, Bougnoux ME, Lortholary O. Mucor irregularis-associated cutaneous mucormycosis: Case report and review. Med Mycol Case Rep. 2014; 6:62-5.

12. Patel A, Bishburg E, Nagarakanti S. Mucormycosis in an HIV-infected renal transplant patient: A case report and review of the literature. Am J Case Rep. 2014; 15:74-8.

13. Park W, Jang M, Hwang E, Han S, Park S, Kim H, Choe M. Allograft mucormycosis due to Rhizopus microsporus in a kidney transplant recipient. Transplant Proc. 2014; 46(2):623-5.

14. Neto FM, Camargo PC, Costa AN, Teixeira RH, Carraro RM, Afonso JE, Jr., Campos SV, Samano MN, Fernandes LM, Abdalla LG et al. Fungal infection by Mucorales order in lung transplantation: 4 case reports. Transplant Proc. 2014; 46(6):1849-51.

15. Navanukroh O, Jitmuang A, Chayakulkeeree M, Ngamskulrungroj P. Disseminated Cunninghamella bertholletiae infection with spinal epidural abscess in a kidney transplant patient: case report and literature review. Transpl Infect Dis. 2014; 16(4):658-65.

16. Hamdi T, Karthikeyan V, Alangaden GJ. Mucormycosis in a renal transplant recipient: case report and comprehensive review of literature. Int J Nephrol. 2014; 2014:950643.

17. Barnajian M, Gioia W, Iordache F, Bergamaschi R. Mucormycosis-induced colon perforation after renal transplantation. Surg Infect (Larchmt). 2014; 15(5):665-6.

18. Turan MN, Tatar E, Yaprak M, Arda B, Kitis O, Metin DY, Hoscoskun C, Toz H. A mucormycosis case presented with orbital apex syndrome and hemiplegia in a renal transplant patient. Int Urol Nephrol. 2013; 45(6):1815-9.

19. Ram R, Swarnalatha G, Naidu GD, Kaligotla DV. Multiple ring enhancing lesions in brain due to disseminated Zygomycosis in a renal transplant recipient. Nephrology (Carlton). 2013; 18(6):479-80.

20. Kwan LP, Choy CB, Chan TM, Suen WS, Yap DY. Successful treatment of pulmonary rhizopus infection with surgical resection and posaconazole in a renal transplant recipient. Nephrology (Carlton). 2013; 18(1):74-5.

21. Kuy S, He C, Cronin DC, 2nd. Renal mucormycosis: a rare and potentially lethal complication of kidney transplantation. Case Rep Transplant. 2013; 2013:915423.

22. Jha R, Gude D, Chennamsetty S, Kotari H. Intracranial hypertension: An unusual presentation of mucormycosis in a kidney transplant recipient. Indian J Nephrol. 2013; 23(2):130-2.

23. Hatahet MH, Narayanan M, Cleaves C, Zreik R. Disseminated mucormycosis in a patient with recent kidney transplantation: a case report and review of the literature. Case Rep Nephrol Urol. 2013; 3(1):58-63.

24. Gupta KL, Joshi K, Bhat A, Kohli HS, Jha V, Sakhuja V. Mucormycosis of the transplanted kidney with renal papillary necrosis. Exp Clin Transplant. 2013; 11(6):554-7.

25. Zhao L, Wang CX, Zhang L, Tu XA, Wang W, Chen Y, Liu LS. Mucormycosis extending from the surgical wound to the transplanted kidney: case report and literature review. Exp Clin Transplant. 2012; 10(4):403-5.

26. Ribeiro LC, Wanke B, da Silva M, Dias LB, Mello R, Canavarros FA, Leite-Jr DP, Hahn RC. Mucormycosis in Mato Grosso, Brazil: a case reports, caused by Rhizopus microsporus var. oligosporus and Rhizopus microsporus var. rhizopodiformis. Mycopathologia. 2012; 173(2-3):187-92.

27. Gupta KL, Joshi K, Kohli HS, Jha V, Sakhuja V. Mucormycosis (zygomycosis) of renal allograft. Clin Kidney J. 2012; 5(6):502-7.

28. Chipde SS, Ranjan P, Lal H, Singh V, Naval R, Marak RS, Prakash A, Bhadoria D, Sharma RK, Kapoor R et al. Isolated renal zygomycosis: novel diagnostic and prognostic criteria with experience of a tertiary care center. Urol Int. 2012; 88(3):282-8.

29. Bhutada K, Borkar SS, Mendiratta DK, Shende VR. Successful treatment of peritonitis by C. bertholletiae in a chronic kidney failure patient on continuous ambulatory peritoneal dialysis after kidney rejection. Singapore Med J. 2012; 53(5):e106-9.

30. Azarpira N, Ashraf MJ, Kazemi K, Khademi B. Rhinomaxillary mucormycosis in a renal transplant recipient: case report. Exp Clin Transplant. 2012; 10(6):605-8.

31. Al Maskari Z, Al Lawatia F. Postrenal transplant renopulmonary zygomycosis with vascular aneurysms responded to surgical treatment and salvage therapy with posaconazole after failure to respond to liposomal amphotericin. Am J Case Rep. 2012; 13:202-5.

32. Kempker J, Sheth A, Kempker R. Cavitary lung lesions in a renal transplant recipient. Clin Infect Dis. 2011; 53(1):59, 94-5.

33. Gupta A, Lal C, Dogra PM, Mahajan S, Agarwal SK. Insulin site wound in a renal allograft recipient. Saudi J Kidney Dis Transpl. 2011; 22(1):134-5.

34. Godara SM, Kute VB, Goplani KR, Gumber MR, Gera DN, Shah PR, Vanikar AV, Trivedi HL. Mucormycosis in renal transplant recipients: predictors and outcome. Saudi J Kidney Dis Transpl. 2011; 22(4):751-6.

35. Colon-Santos E, Gonzalez-Ramos M, Bertran-Pasarell J, Rodriguez-Vega G, Almira-Suarez M, Velez-Rosario R. Disseminated nocardiosis masking an atypical zygomycosis presentation in a kidney transplant recipient. Transpl Infect Dis. 2011; 13(4):380-4.

36. Woo PC, Lau SK, Ngan AH, Tung ET, Leung SY, To KK, Cheng VC, Yuen KY. Lichtheimia hongkongensis sp. nov., a novel Lichtheimia spp. associated with rhinocerebral, gastrointestinal, and cutaneous mucormycosis. Diagn Microbiol Infect Dis. 2010; 66(3):274-84.

37. Sun HY, Forrest G, Gupta KL, Aguado JM, Lortholary O, Julia MB, Safdar N, Patel R, Kusne S, Singh N. Rhino-orbital-cerebral zygomycosis in solid organ transplant recipients. Transplantation. 2010; 90(1):85-92.

38. Hofman V, Dhouibi A, Butori C, Padovani B, Gari-Toussaint M, Garcia-Hermoso D, Baumann M, Venissac N, Cathomas G, Hofman P. Usefulness of molecular biology performed with formaldehyde-fixed paraffin embedded tissue for the diagnosis of combined pulmonary invasive mucormycosis and aspergillosis in an immunocompromised patient. Diagn Pathol. 2010; 5:1.

39. Alexander BD, Schell WA, Siston AM, Rao CY, Bower WA, Balajee SA, Howell DN, Moore ZS, Noble-Wang J, Rhyne JA et al. Fatal Apophysomyces elegans infection transmitted by deceased donor renal allografts. Am J Transplant. 2010; 10(9):2161-7.

40. Varma PP, Hooda AK, Badwal S. Laryngeal stridor and myocardial infarction in a renal transplant recipient. Natl Med J India. 2009; 22(2):70-1.

41. Spellberg B, Andes D, Perez M, Anglim A, Bonilla H, Mathisen GE, Walsh TJ, Ibrahim AS. Safety and outcomes of open-label deferasirox iron chelation therapy for mucormycosis. Antimicrob Agents Chemother. 2009; 53(7):3122-5.

42. Li JY, Yong TY, Jurisevic CA, Russ GR, Grove DI, Coates PT, Disney AP. Successful treatment of pulmonary mucormycosis in a renal transplant recipient with limited pulmonary reserve by combined medical and surgical therapy. Heart Lung Circ. 2009; 18(3):226-8.

43. Lakshminarayana G, Rajesh R, Kurian G, Unni VN. Zygomycosis in a renal allograft recipient. Indian J Nephrol. 2009; 19(1):30-3.

44. Zhan HX, Lv Y, Zhang Y, Liu C, Wang B, Jiang YY, Liu XM. Hepatic and renal artery rupture due to Aspergillus and Mucor mixed infection after combined liver and kidney transplantation: a case report. Transplant Proc. 2008; 40(5):1771-3.

45. Yassin MA, Taj-Aldeen SJ, Khan FY, Errayes M, Aref E. Rhino-orbital zygomycosis secondary to Rhizopus oryzae in a renal transplant recipient successfully treated with liposomal amphotericin B. Chang Gung Med J. 2008; 31(4):407-11.

46. Stelzmueller I, Lass-Floerl C, Geltner C, Graziadei I, Schneeberger S, Antretter H, Mueller L, Zelger B, Singh N, Pruett TL et al. Zygomycosis and other rare filamentous fungal infections in solid organ transplant recipients. Transpl Int. 2008; 21(6):534-46.

47. Nouri-Majalan N, Moghimi M. Skin mucormycosis presenting as an erythema-nodosum-like rash in a renal transplant recipient: a case report. J Med Case Rep. 2008; 2:112.

48. Nalmas S, Bishburg E, Goldstein C. Mucormycosis in a transplanted kidney. Transpl Infect Dis. 2008; 10(4):269-71.

49. Mysorekar VV, Rao SG. Cytomegalovirus pneumonia with pulmonary mucormycosis. Indian J Pathol Microbiol. 2008; 51(2):294-5.

50. Miladipour A, Ghanei E, Nasrollahi A, Moghaddasi H. Successful treatment of mucormycosis after kidney transplantation. Iran J Kidney Dis. 2008; 2(3):163-6.

51. Madiwale C, Murlidharan P, Hase NK. Recurrence of primary hyperoxaluria: an avoidable catastrophe following kidney transplant. J Postgrad Med. 2008; 54(3):206-8.

52. Bakr A, Wafa E, Fouda A, Elagroudy A, Gheith O, Sobh M, Shokeir A, Ghoneim M. Successful treatment of mucormycosis in a renal allograft recipient. Clin Exp Nephrol. 2008; 12(3):207-10.

53. Uckay I, Chalandon Y, Sartoretti P, Rohner P, Berney T, Hadaya K, van Delden C. Invasive zygomycosis in transplant recipients. Clin Transplant. 2007; 21(4):577-82.

54. Tomazic J, Pirs M, Matos T, Ferluga D, Lindic J. Multiple infections after commercial renal transplantation in India. Nephrol Dial Transplant. 2007; 22(3):972-3.

55. Tayyebi N, Amouian S, Mohamadian N, Rahimi HR. Renal allograft mucormycosis: report of two cases. Surg Infect (Larchmt). 2007; 8(5):535-8.

56. Saikia UN, Jain D, Joshi K, Lal A, Sakhuja V. Disseminated zygomycosis presenting as thyroid abscess in a renal allograft recipient. Nephrol Dial Transplant. 2007; 22(2):641-4.

57. Pradhan A, Gadela S, Kumar RS, Kalghatghi A, Pradhan S. To bite the bullet of early graft nephrectomy: a case report. Transplant Proc. 2007; 39(5):1664-5.

58. Harada AS, Lau W. Successful treatment and limb salvage of mucor necrotizing fasciitis after kidney transplantation with posaconazole. Hawaii Med J. 2007; 66(3):68-71.

59. Forrest GN, Mankes K. Outcomes of invasive zygomycosis infections in renal transplant recipients. Transpl Infect Dis. 2007; 9(2):161-4.

60. Chacko B, David VG, Tamilarasi V, Deepti AN, John GT. Pulmonary mucormycosis in a nondiabetic renal allograft recipient successfully managed by medical therapy alone. Transplantation. 2007; 83(12):1656-7.

61. Aslani J, Eizadi M, Kardavani B, Khoddami-Vishteh HR, Nemati E, Hoseini SM, Einollahi B. Mucormycosis after kidney transplantations: report of seven cases. Scand J Infect Dis. 2007; 39(8):703-6.

62. Prasad N, Ram R, Satti Reddy V, Dakshinamurty KV. Non-fatal gastric mucormycosis in a renal transplant patient and review of the literature. Transpl Infect Dis. 2006; 8(4):237-41.

63. Monecke S, Hochauf K, Gottschlich B, Ehricht R. A case of peritonitis caused by Rhizopus microsporus. Mycoses. 2006; 49(2):139-42.

64. Liapis CD, Petrikkos GL, Paraskevas KI, Skiada A, Nikolaou AC, Tzortzis G, Kostakis AG. External Iliac artery stent mucormycosis in a renal transplant patient. Ann Vasc Surg. 2006; 20(2):253-7.

65. Almyroudis NG, Sutton DA, Linden P, Rinaldi MG, Fung J, Kusne S. Zygomycosis in solid organ transplant recipients in a tertiary transplant center and review of the literature. Am J Transplant. 2006; 6(10):2365-74.

66. Mekeel KL, Hemming AW, Reed AI, Matsumoto T, Fujita S, Schain DC, Nelson DR, Dixon LR, Fujikawa T. Hepatic mucormycosis in a renal transplant recipient. Transplantation. 2005; 79(11):1636.

67. Echo A, Hovsepian RV, Shen GK. Localized cecal zygomycosis following renal transplantation. Transpl Infect Dis. 2005; 7(2):68-70.

68. Alkhunaizi AM, Amir AA, Al-Tawfiq JA. Invasive fungal infections in living unrelated renal transplantation. Transplant Proc. 2005; 37(7):3034-7.

69. Ahmad M. Graft mucormycosis in a renal allograft recipient. J Nephrol. 2005; 18(6):783-6.

70. Sehgal A, Raghavendran M, Kumar D, Srivastava A, Dubey D, Kumar A. Rhinocerebral mucormycosis causing basilar artery aneurysm with concomitant fungal colonic perforation in renal allograft recipient: a case report. Transplantation. 2004; 78(6):949-50.

71. Quinio D, Karam A, Leroy JP, Moal MC, Bourbigot B, Masure O, Sassolas B, Le Flohic AM. Zygomycosis caused by Cunninghamella bertholletiae in a kidney transplant recipient. Med Mycol. 2004; 42(2):177-80.

72. Crompton JA, Alexander D, Somerville T, Shihab FS. Lipid-based amphotericin in pulmonary zygomycosis: safety and efficacy of high exposure in a renal allograft recipient. Transpl Infect Dis. 2004; 6(4):183-7.

73. Serna JH, Wanger A, Dosekun AK. Successful treatment of mucormycosis peritonitis with liposomal amphotericin B in a patient on long-term peritoneal dialysis. Am J Kidney Dis. 2003; 42(3):E14-7.

74. Minz M, Sharma A, Kashyap R, Udgiri N, Heer M, Kumar V, Vaiphei K. Isolated renal allograft arterial mucormycosis: an extremely rare complication. Nephrol Dial Transplant. 2003; 18(5):1034-5.

75. Ladurner R, Brandacher G, Steurer W, Schneeberger S, Bosmuller C, Freund MC, Kreczy A, Konigsrainer A, Margreiter R. Lessons to be learned from a complicated case of rhino-cerebral mucormycosis in a renal allograft recipient. Transpl Int. 2003; 16(12):885-9.

76. Zhang R, Zhang JW, Szerlip HM. Endocarditis and hemorrhagic stroke caused by Cunninghamella bertholletiae infection after kidney transplantation. Am J Kidney Dis. 2002; 40(4):842-6.

77. Severo LC, Oliveira FD, Dreher R, Teixeira PZ, Porto ND, Londero AT. Zygomycosis: A report of eleven cases and a review of the Brazilian literature. Rev Iberoam Micol. 2002; 19(1):52-6.

78. Jimenez C, Lumbreras C, Paseiro G, Loinaz C, Romano DR, Andres A, Aguado JM, Morales JM, del Palacio A, Garcia I et al. Treatment of mucor infection after liver or pancreas-kidney transplantation. Transplant Proc. 2002; 34(1):82-3.

79. Jimenez C, Lumbreras C, Aguado JM, Loinaz C, Paseiro G, Andres A, Morales JM, Sanchez G, Garcia I, del Palacio A et al. Successful treatment of mucor infection after liver or pancreas-kidney transplantation. Transplantation. 2002; 73(3):476-80.

80. Bhowmik D, Dinda AK, Khilnani GC, Mahajan S, Gupta S, Agarwal SK, Tiwari SC, Dash SC. Pulmonary mucormycosis in a diabetic renal transplant patient. Indian J Chest Dis Allied Sci. 2002; 44(4):275-7.

81. Tinmouth J, Baker J, Gardiner G. Gastrointestinal mucormycosis in a renal transplant patient. Can J Gastroenterol. 2001; 15(4):269-71.

82. Sohail MA, Al Khabori M, Hyder J, Verma A. Acute fulminant fungal sinusitis: clinical presentation, radiological findings and treatment. Acta Trop. 2001; 80(2):177-85.

83. Lee E, Vershvovsky Y, Miller F, Waltzer W, Suh H, Nord EP. Combined medical surgical therapy for pulmonary mucormycosis in a diabetic renal allograft recipient. Am J Kidney Dis. 2001; 38(6):E37.

84. Ju JH, Park HS, Shin MJ, Yang CW, Kim YS, Choi YJ, Song HJ, Kim SW, Chung IS, Bang BK. Successful treatment of massive lower gastrointestinal bleeding caused by mixed infection of cytomegalovirus and mucormycosis in a renal transplant recipient. Am J Nephrol. 2001; 21(3):232-6.

85. Chkhotua A, Yussim A, Tovar A, Weinberger M, Sobolev V, Bar-Nathan N, Shaharabani E, Shapira Z, Mor E. Mucormycosis of the renal allograft: case report and review of the literature. Transpl Int. 2001; 14(6):438-41.

86. Bakshi NA, Volk EE. Pulmonary mucormycosis diagnosed by fine needle aspiration cytology. A case report. Acta Cytol. 2001; 45(3):411-4.

87. Stoebner PE, Gaspard C, Mourad G, Beraud JJ, Meynadier J, Meunier L. Fulminant mucormycosis in a renal transplant recipient. Acta Derm Venereol. 2000; 80(4):305.

88. Demirag A, Elkhammas EA, Henry ML, Davies EA, Pelletier RP, Bumgardner GL, Dorner B, Ferguson RM. Pulmonary Rhizopus infection in a diabetic renal transplant recipient. Clin Transplant. 2000; 14(1):8-10.

89. Adriaenssens K, Jorens PG, Meuleman L, Jeuris W, Lambert J. A black necrotic skin lesion in an immunocompromised patient. Diagnosis: cutaneous mucormycosis. Arch Dermatol. 2000; 136(9):1165-70.

90. Seiff SR, Choo PH, Carter SR. Role of local amphotericin B therapy for sino-orbital fungal infections. Ophthal Plast Reconstr Surg. 1999; 15(1):28-31.

91. Gupta KL, Khullar DK, Behera D, Radotra BD, Sakhuja V. Pulmonary mucormycosis presenting as fatal massive haemoptysis in a renal transplant recipient. Nephrol Dial Transplant. 1998; 13(12):3258-60.

92. Apaydin S, Ataman R, Cansiz H, Serdengecti K, Ozturk R, Dervisoglu S, Erek E, Ulku U. Rhinocerebral mucormycosis in a kidney transplant recipient. Nephron. 1998; 79(1):117-8.

93. Martinez EJ, Cancio MR, Sinnott JTt, Vincent AL, Brantley SG. Nonfatal gastric mucormycosis in a renal transplant recipient. South Med J. 1997; 90(3):341-4.

94. Latif S, Saffarian N, Bellovich K, Provenzano R. Pulmonary mucormycosis in diabetic renal allograft recipients. Am J Kidney Dis. 1997; 29(3):461-4.

95. Winkler S, Susani S, Willinger B, Apsner R, Rosenkranz AR, Potzi R, Berlakovich GA, Pohanka E. Gastric mucormycosis due to Rhizopus oryzae in a renal transplant recipient. J Clin Microbiol. 1996; 34(10):2585-7.

96. Stas KJ, Louwagie PG, Van Damme BJ, Coosemans W, Waer M, Vanrenterghem YF. Isolated zygomycosis in a bought living unrelated kidney transplant. Transpl Int. 1996; 9(6):600-2.

97. Nampoory MR, Khan ZU, Johny KV, Constandi JN, Gupta RK, Al-Muzairi I, Samhan M, Mozavi M, Chugh TD. Invasive fungal infections in renal transplant recipients. J Infect. 1996; 33(2):95-101.

98. Naguib MT, Huycke MM, Pederson JA, Pennington LR, Burton ME, Greenfield RA. Apophysomyces elegans infection in a renal transplant recipient. Am J Kidney Dis. 1995; 26(2):381-4.

99. Mitwalli A, Malik GH, al-Wakeel J, Abu Aisha H, al-Mohaya S, al-Jaser A, Assaf H, el Gamal H. Mucormycosis of the graft in a renal transplant recipient. Nephrol Dial Transplant. 1994; 9(6):718-20.

100. Andrews PA, Abbs IA, Koffman CG, Ogg CS, Williams DG. Mucormycosis in transplant recipients: possible case-case transmission and potentiation by cytomegalovirus. Nephrol Dial Transplant. 1994; 9(8):1194-6.

101. Norden G, Bjorck S, Persson H, Svalander C, Li XG, Edebo L. Cure of zygomycosis caused by a lipase-producing Rhizopus rhizopodiformis strain in a renal transplant patient. Scand J Infect Dis. 1991; 23(3):377-82.

102. Das T, Gupta A, Sakhuja V, Gupta KL, Minz M, Chugh KS. Ocular complications in renal allograft recipients. Nephrol Dial Transplant. 1991; 6(9):649-55.

103. Hsu J, Clayman JA, Geha AS. Survival of a recipient of renal transplantation after pulmonary phycomycosis. Ann Thorac Surg. 1989; 47(4):617-9.

104. Hamdy NA, Andrew SM, Shortland JR, Boletis J, Raftery AT, Kanis JA, Brown CB. Fatal cardiac zygomycosis in a renal transplant patient treated with desferrioxamine. Nephrol Dial Transplant. 1989; 4(10):911-3.

105. Narang AK, Dina TS. Cerebral mucormycosis: a case report. Comput Med Imaging Graph. 1988; 12(4):259-62.

106. Kaplan AH, Poza-Juncal E, Shapiro R, Stapleton JT. Cure of mucormycosis in a renal transplant patient receiving ciclosporin with maintenance of immunosuppression. Am J Nephrol. 1988; 8(2):139-42.

107. Morduchowicz G, Shmueli D, Shapira Z, Cohen SL, Yussim A, Block CS, Rosenfeld JB, Pitlik SD. Rhinocerebral mucormycosis in renal transplant recipients: report of three cases and review of the literature. Rev Infect Dis. 1986; 8(3):441-6.

108. Tuder RM. Myocardial infarct in disseminated mucormycosis: case report with special emphasis on the pathogenic mechanisms. Mycopathologia. 1985; 89(2):81-8.

109. Carbone KM, Pennington LR, Gimenez LF, Burrow CR, Watson AJ. Mucormycosis in renal transplant patients--a report of two cases and review of the literature. Q J Med. 1985; 57(224):825-31.

110. West BC, Kwon-Chung KJ, King JW, Grafton WD, Rohr MS. Inguinal abscess caused by Rhizopus rhizopodiformis: successful treatment with surgery and amphotericin B. J Clin Microbiol. 1983; 18(6):1384-7.

111. Peterson PK, Dahl MV, Howard RJ, Simmons RL, Najarian JS. Mucormycosis and cutaneous histoplasmosis in a renal transplant recipient. Arch Dermatol. 1982; 118(4):275-7.

112. Gribetz AR, Chuang MT, Burrows L, Teirstein AS. Rhizopus lung abscess in renal transplant patient successfully treated by lobectomy. Chest. 1980; 77(1):102-4.

113. Fisher J, Tuazon CU, Geelhoed GW. Mucormycosis in transplant patients. Am Surg. 1980; 46(5):315-22.

114. Cohen SG, Greenberg MS. Rhinomaxillary mucormycosis in a kidney transplant patient. Oral Surg Oral Med Oral Pathol. 1980; 50(1):33-8.

115. Henriquez M, Levy R, Raja RM, Kramer MS, Rosenbaum JL. Mucormycosis in a renal transplant recipient with successful outcome. JAMA. 1979; 242(13):1397-9.

116. Ramon Y, Oberman M, Horowitz I, Freedman A. Extensive maxillary sequestration resulting from rhinocerebral mucormyocosis. J Oral Surg. 1977; 35(12):989-91.

117. Wilson CB, Siber GR, O'Brien TF, Morgan AP. Phycomycotic gangrenous cellulitis. A report of two cases and a review of the literature. Arch Surg. 1976; 111(5):532-8.

118. Thorsteinsson SB, Musher DM, Ortiz LR, Wolfe KS, Weinman EJ, Schloeder FX. Phycomycosis in a renal transplant recipient. Tex Med. 1976; 72(4):71-5.

119. Braf ZF, Altmann G, Ostfeld E. Fungal infections after renal transplantation. Isr J Med Sci. 1976; 12(7):674-7.

120. Zwas ST, Czerniak P. Head and brain scan findings in rhinocerebral mucormycosis: case report. J Nucl Med. 1975; 16(10):925-7.

121. Hammer GS, Bottone EJ, Hirschman SZ. Mucormycosis in a transplant recipient. Am J Clin Pathol. 1975; 64(3):389-98.

122. Stevens KM, Newell RC, Bergstrom L. Mucormycosis in a patient receiving azathioprine. Arch Otolaryngol. 1972; 96(3):250-1.

123. Haim S, Better OS, Lichtig C, Erlik D, Barzilai A. Rhinocerebral mucormycosis following kidney transplantation. Isr J Med Sci. 1970; 6(5):646-9.
